# Changes of Endophytic Bacterial Community in Mature Leaves of *Prunus laurocerasus* L. during the Seasonal Transition from Winter Dormancy to Vegetative Growth

**DOI:** 10.3390/plants11030417

**Published:** 2022-02-03

**Authors:** Jaroslav Michalko, Juraj Medo, Peter Ferus, Jana Konôpková, Dominika Košútová, Peter Hoťka, Marek Barta

**Affiliations:** 1 Mlynany Arboretum, Institute of Forest Ecology, Slovak Academy of Sciences, 951-52 Slepcany, Slovakia; peter.ferus@savba.sk (P.F.); jana.konopkova@savba.sk (J.K.); dominika.bosiakova@savba.sk (D.K.); peter.hotka@savba.sk (P.H.); 2 Institute of Biotechnology, Faculty of Biotechnology and Food Sciences, Slovak University of Agriculture in Nitra, 949-76 Nitra, Slovakia; juraj.medo@uniag.sk; 3 Department of Botany and Genetics, Faculty of Natural Sciences, Constantine the Philosopher University in Nitra, 949-74 Nitra, Slovakia; 4 Department of Plant Pathology and Mycology, Institute of Forest Ecology, Slovak Academy of Sciences, 949-01 Nitra, Slovakia; marek.barta@savba.sk

**Keywords:** cherry laurel, plant microbiome, leaf endosphere, 16S rRNA gene metabarcoding, biodiversity

## Abstract

Diverse communities of bacterial endophytes inhabit plant tissues, and these bacteria play important roles for plant growth and health. Cherry laurel (*Prunus laurocerasus* L.) is a broadleaf evergreen shrub that is widely grown in temperate zones for its ornamental and medicinal properties, however virtually nothing is known about its associated bacterial community. In this study, we analysed the matured one-year-old leaves of this plant using Illumina-based 16S rRNA gene metabarcoding to reveal the community structure of endophytic bacteria and understand its shifts during the seasonal transition from winter dormancy to a spring vegetative state. The overall community was composed of four dominant phyla (*Proteobacteria*, *Actinobacteria*, *Firmicutes*, *Bacteroidetes*). *Corynebacterium*, *Acinetobacter*, and *Chryseobacterium* genera were the most prevalent bacteria, comprising 13.3%, 6.9%, and 6.8% of the amplicon sequence variants (ASVs), respectively. The ASV richness and diversity increased significantly in May as compared to other sampling months (February, March, and April). We observed high variation in the overall community structure of endophytic bacteria among collection dates. The variation was only reflected by a few core community members, suggesting that the changes of the endophytic community during winter/spring seasonal transition are mostly associated with the less abundant community members. We identified biomarker taxa for late winter, mid spring, and late spring collection dates. This study is the first one to report on the diversity and composition of bacterial endophytes in the leaves of cherry laurel and its shifts across the dormancy-to-vegetative seasonal transition.

## 1. Introduction

In nature, all plants are inhabited by a diverse spectrum of associated microorganisms which colonize their surface as well as the inner tissues [1,2,3]. Bacterial endophytes represent a subset of plant-associated microbial communities. They inhabit healthy plant tissues but do not lead to pathogenic reactions [4,5,6] and play important roles in plant growth promotion, plant tolerance against herbivores, pathogens or abiotic stress, and in phytoremediation [3,7,8,9,10,11,12,13,14]. Plant, animal, and human pathogenic bacteria may also survive asymptomatically within plant tissues [15]. Exploration of the diversity and community composition of endophytic bacteria has been traditionally conducted, applying culture-dependent microbiological approaches with a special emphasis on the root plant growth-promoting bacteria of cultivated plants including rapeseed [16], potato [17], and sugarcane [18] because of their large contribution to plant nutrient intake as well as to the high diversity of soil bacteria. While culturing methods are able to uncover specific roles of bacteria, it has been estimated that only 1% of bacterial communities are culturable [19,20]. Modern, culture-independent methods possess the potential to analyse whole microbial communities by DNA profiling or 16S rRNA gene metabarcoding. NGS technology has been used to characterize the core subsets of endophytic bacterial communities of several agronomically and medicinally important woody plant species [3,21,22,23,24,25,26,27,28,29,30,31]. In these studies, great diversity of bacterial endophytic communities, and different factors shaping the community composition of endophytic microbiomes, have been identified, such as plant genotype, developmental stage, physiological status, tissue, habitat, season, environmental conditions, and the disease status of the plant host. One basic question that has rarely been addressed by molecular approaches for the phyllosphere is whether there are seasonal or annual patterns in its bacterial community structure and, specifically, how it is changed during seasonal transitions from winter dormancy to vegetative period. Seasonal patterns have been analysed and confirmed mostly for deciduous trees from leaf emergence to leaf fall, e.g., in poplar trees [32], inside tree branches [33], in maple tree sap [34], or in buds of Scots pine [35]. In this respect, evergreen trees may represent a different environment in that leaves are present year-round so that temporal variation in phyllosphere communities may not be as closely tied to successional changes with leaf development. The longer leaf lifespan of evergreen woody perennials could also allow for the establishment of a more diverse and stable community of core microbes over time as compared to deciduous trees or a herbaceous plant with an annual lifespan. There is still an open question as to whether the leaves of broad-leafed evergreen perennials store endophytic communities from previous season or whether they are recolonized each year. Especially interesting, therefore, are fluctuations in the bacterial community in mature leaves during the seasonal transition from winter dormancy to vegetative growth. Very limited information is available in literature on this topic for broad-leafed evergreen perennials. There is only a single study [36] in which the authors examined seasonal variations in the phyllosphere bacterial community of evergreen magnolia trees (*Magnolia grandiflora*) using denaturing gradient gel electrophoresis of 16S rRNA amplicons.

Cherry laurel (*P. laurocerasus* L., syn. *Laurocerasus officinalis* M. Roem.; *Rosaceae*) is a broadleaf evergreen shrub growing up to 6 m. It was introduced from Eastern Europe and Western Asia into different parts of the world, where it is mass cultivated as an ornamental garden and hedge plant. It belongs to one of the most important hardy nursery stock species. A high concentration (1% to 2.5%) of medicinally important α-hydroxymandelonitrile derivatives of cyanogenic glycosides was found in cherry laurel leaves, fruits, and seeds such as prunasin, sambunigrin, and amygdalin [37], which possess pharmacological activities [38]. Cherry laurel fruits are used in traditional medicine for stomach ulcers, digestive system complaints, bronchitis, eczemas, haemorrhoids, and as diuretic, antipyretic, and as analgesic agents [39].

Broadleaf evergreen plants such as cherry laurel represent good model systems to continuously monitor the fluctuations of residing endophytic microbiome in response to changing climate conditions also outside the typical growing season, which might have important implications for the host plant biology and its ability to tolerate biotic and abiotic stress. In our study, we sought to characterize for the first time the nature of the bacterial community in the leaf endosphere of a broad-leafed evergreen shrub cherry laurel using 16S rRNA gene metabarcoding. More importantly, we examined how the diversity and community composition of endophytic bacteria in mature leaves of cherry laurel change during the transition period from winter dormancy into a vegetation state. We hypothesized that, during the winter months, only a minor number of core bacterial species can survive within the leaves of cherry laurel and, with the onset of higher temperatures during the spring, the richness and diversity of bacterial endophytes gradually increase.

## 2. Results

### 2.1. Bacterial Community Found in Cherry Laurel Leaves

Sequencing of the amplicon libraries resulted in a total of 386,915 raw reads prior to quality checking and the assigning of the reads to the respective sample. After applying all of the quality filters, removing chimeras, exclusion of chloroplast (0.12% of reads), and mitochondrial ASVs (31.1% reads), a total of 154,621 high-quality reads were recovered from all 60 samples (an average of 2577 sequences per sample), with a range of 889–10,400 sequences. These sequence data have been submitted to the GenBank databases under BioProject accession No. PRJNA609065. Among all samples, 769 amplicon sequencing variants (ASVs) detected by DADA2 algorithm were used for analysis. Rarefaction curves for all samples are shown in Figure S1.

At the domain level, bacterial and archaeal sequences were represented in the dataset, with their relative abundances and ASV numbers reaching 97% and 751 ASVs for Bacteria and 3% and 18 ASVs for Archaea, respectively. Archaeal ASVs were represented by a single genus, Nitrososphaera, of the phylum Thaumarchaeota. Among bacteria, at the phylum level, the phyla of Proteobacteria (37.4%), Actinobacteria (22.5%), Firmicutes (21.8%), and Bacteroidetes (13.4%) were the most abundant (Table 2). Of the Proteobacteria-affiliated classes, the majority was represented by Gammaproteobacteria (23.0%). Alphaproteobacteria and Betaproteobacteria were less common (6.1% and 8.2%, respectively). In addition, the class Actinobacteria (22.5%) of the phylum Actinobacteria and the class Bacilli (16.8%) of the phylum Firmicutes were in dominant positions. At family level, the most abundant populations were the families Corynebacteriaceae (Actinobacteria, 13.3%), Flavobacteriaceae (Flavobacteriia, 10.5%), Moraxeallaceae (Gammaproteobacteria, 9.5%), and Streptococcaceae (Bacilli, 9.1%). At the genus level, Corynebacterium (Actinobacteria; 13.3%, 23 ASVs) dominated the endophytic bacterial community of P. laurocerasus leaves, followed by Acinetobacter (Gammaproteobacteria; 6.9%, 17 ASVs) and Chryseobacterium (Flavobacteria; 6.8%, 8 ASVs). In addition, Lactococcus, Pseudomonas, Staphylococcus, Streptococcus, and Stenotrophomonas were also abundant genera, with relative abundance greater than 3%.

### 2.2. Community Composition of Leaf Endophytic Bacteria among Different Collection Dates

**Figure 1:**
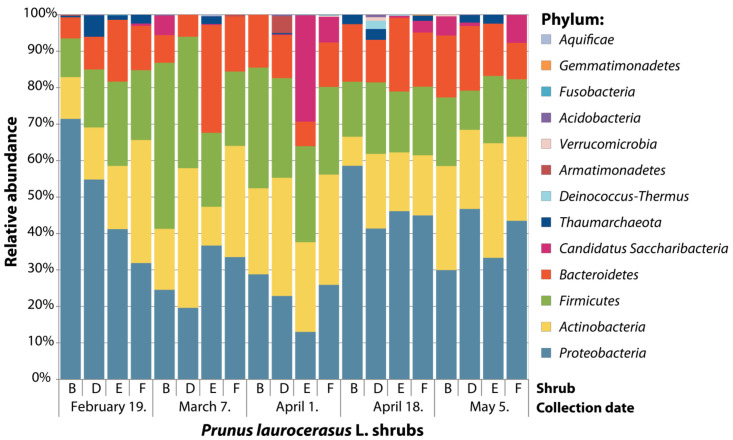
Phylum distribution of the amplicon sequencing variants (ASVs). Relative sequence abundance (RA) of prokaryotic phyla associated with leaf endosphere. Data for all leaf samples collected from the same shrub on the same collection date were pooled together. RA of phyla was calculated as the proportion of sequences belonging to particular phylum of all 16S rRNA sequences recovered from all samples.

The predominant community members in leaf endophytic prokaryotic communities associated with mature cherry laurel leaves were largely consistent among the five collection dates at the phylum level (Figure 1). However, the relative abundance of predominant bacterial and archaeal phyla was highly variable among collection dates as well as among individual P. laurocerasus shrubs. Minor contributing phyla comprised less than 8% of total sequences. The only exception was the candidate Saccharibacteria lineage, which comprised almost 30% of the sequences in a single P. laurocerasus shrub sampled on 1 April.

**Figure 2:**
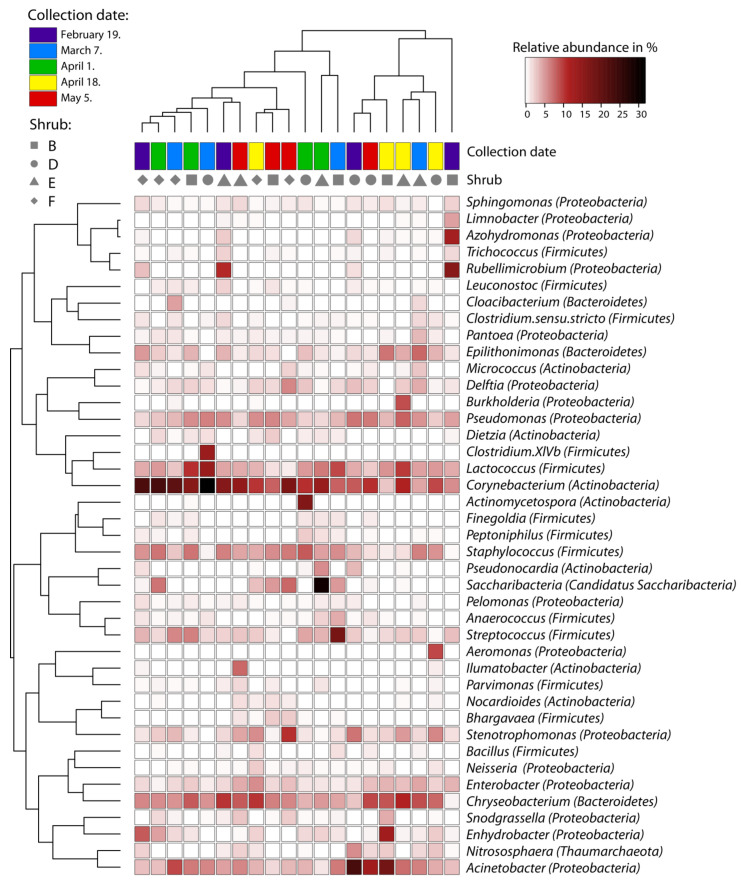
Heatmap of relative abundances of the top 41 prokaryotic genera associated with mature leaves of individual Prunus laurocerasus shrubs at different collection dates. Data for all leaf samples collected from the same shrub on the same collection date were pooled together. Rows are bacterial genera, and columns are individual shrubs. Color scale indicate taxa with a higher (darker colors) or lower (brighter colors) relative sequence abundance in each sample. Only those genera with a relative sequence abundance of 2% or higher in at least one shrub were included.

At the genus level, the relative abundance of dominant bacteria and archaea varied greatly among shrubs, as shown by the heatmap in Figure 2, but also among individual samples (i.e., leaves, data not shown) with no clear clustering into groups based on collection dates or shrubs. We further analysed the diversity and community composition into more detail.

### 2.3. Temporal Variation in Diversity of Mature Leaf Endophytic Bacteria

**Figure 3:**
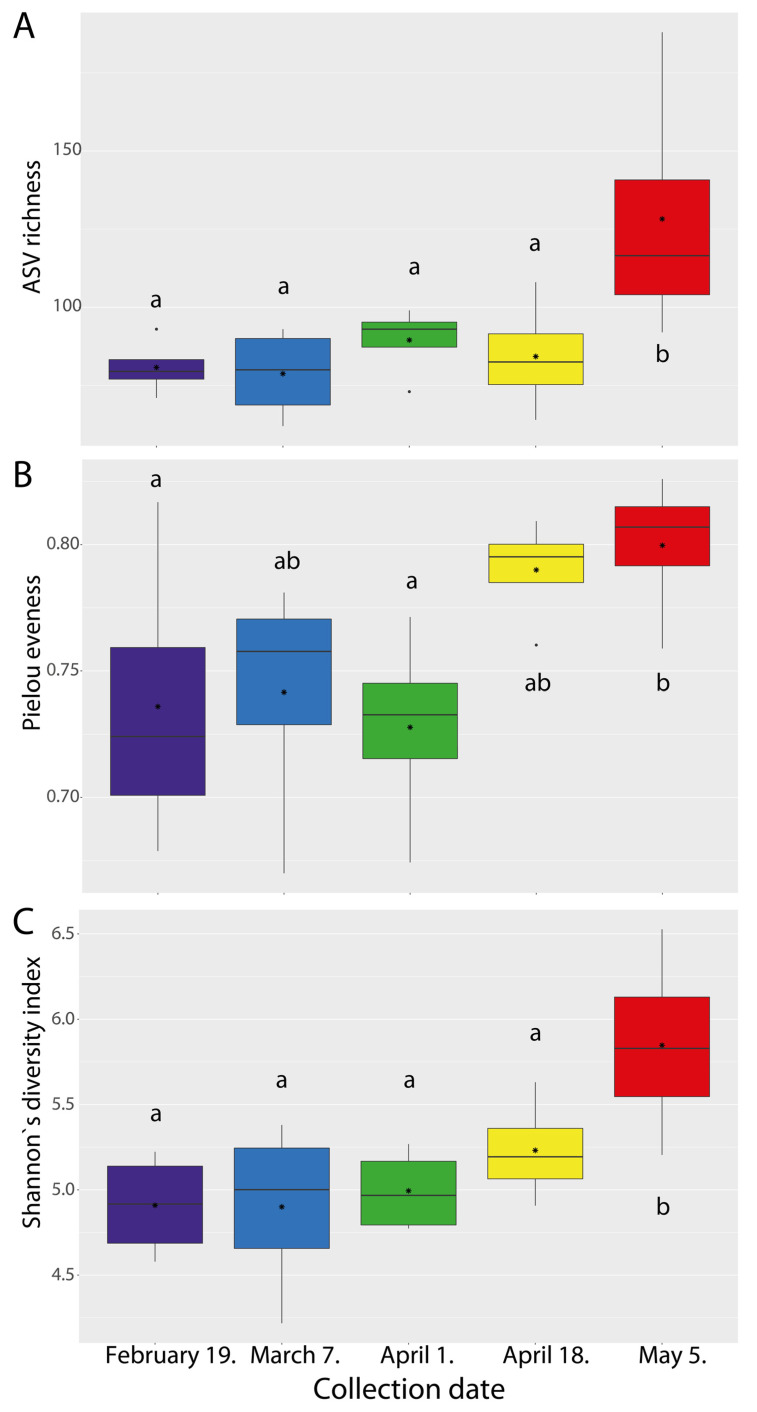
Alpha diversity estimates of bacterial communities: (**A**) ASV richness estimates (number of observed amplicon sequencing variants; ASVs). (**B**) Pielou´s evenness estimates. (**C**) Shannon’s diversity indices. Box plots display the first (25%) and third (75%) quartiles, the median, the average (•), the maximum and minimum observed values, and extreme values (·) within each data set. Data were analysed by means of one-way ANOVAs and Tukey´s Honestly Significant Difference (HSD) post hoc comparisons. Significant differences (*p* < 0.05) across sampling months are indicated with lowercase letters.

Bacterial community richness, evenness (Pielou’s), and alpha diversity (Shannon) index values were compared among five collection dates of cherry laurel mature leaves at the amplicon sequencing variant (ASV) level (Figure 3). The significance levels of ANOVA analysis for ASV richness, Pielou´s evenness, and Shannon diversity were *p* = 0.038, *p* = 0.009, and *p* = 0.024, respectively. ASV richness was significantly higher in samples taken on 4 May as compared to 19 February, 7 March, 1 April, and 18 April samples (Figure 3A). For evenness estimates, we observed in Figure 3B significant differences in the earliest-winter and latest spring collection dates, as well as between 1 April and 4 May. In contrast, diversity estimates showed higher diversity for samples taken in May as compared to samples taken on 19 February, 7 March, 1 April, and 18 April (Figure 3C).

### 2.4. Community Structure of Leaf Endophytic Bacteria among Collection Dates during Seasonal Transition from Winter to Spring

**Figure 4:**
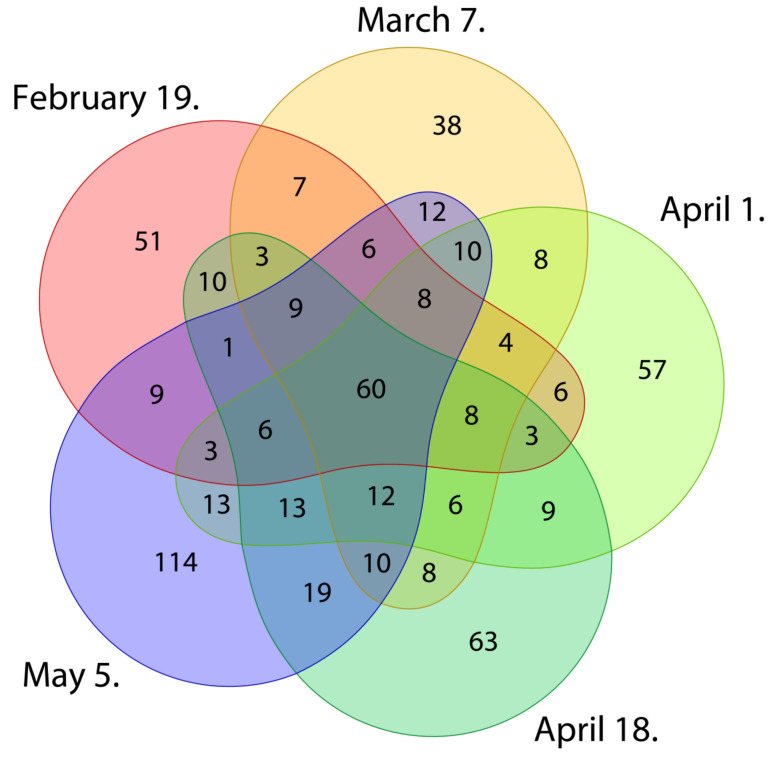
The amplicon sequencing variant (ASV) level of endophytic bacteria distribution in mature P. laurocerasus leaves across the collection dates. Venn diagram shows the number and relative abundance (%) of ASVs shared and unique among different sampling months.

There were several bacterial ASVs shared across the sampling months in association with mature *P. laurocerasus* leaves, and 60 out of 769 bacterial ASVs were shared across all sampling dates (Figure 4). Around 15% (114) of all ASVs were found exclusively in May samples, followed by 63 ASVs in the second April collection date. For other collection dates, numbers of exclusive ASVs were lower that the number of ASVs shared by all collection dates. The amount of ASVs shared in the warmer months (April and May; average daily temperature ±SE = 12.6 ± 0.5 °C; Figure S2) was generally higher than shared ASVs in colder months (February and March; average daily temperature ±SE = 5.0 ± 0.3 °C).

**Figure 5:**
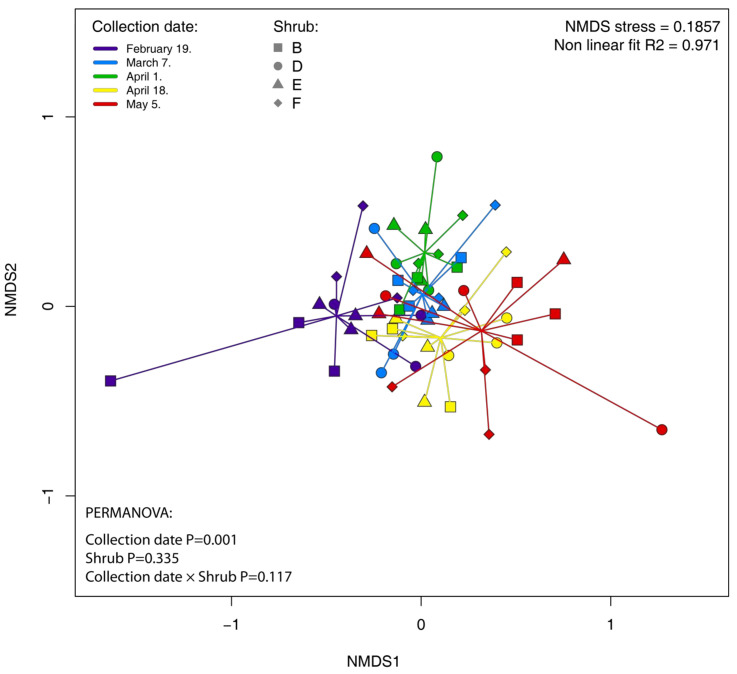
Nonmetric Multi-Dimensional Scaling (NMDS) of bacterial communities by individual shrubs and collection dates based on UniFrac distances. The different colors indicate the leaf collection dates; the shapes indicate shrubs, and each point represents an individual leaf.

The overall similarity of the bacterial community structure among samples is displayed using nonmetric multidimensional scaling (NMDS) based on UniFrac distances (Figure 5). Samples from different collection dates were clustered into overlayed clusters. Nevertheless, permutational multivariate analysis of variance (PERMANOVA) using UniFrac distances shows that collection date is a significant factor in the distances of the samples (*p* = 0.001; Figure 5). We did not observe the effect of individual shrubs on the bacterial community structure (*p* > 0.05), and the variability of bacterial communities among individual leaves was high. Moreover, we did not confirm the effect of the interaction between shrubs and collection dates (*p* > 0.05).

Permutation test for the homogeneity of multivariate dispersion did not find significant differences among dispersions in collection dates. Subsequent pairwise comparisons of collection dates using PERMANOVA (Table 1) showed significant differences between leaf collection dates (Table 1). Moreover, an analysis of similarity ANOSIM confirmed these results (Table S1). Most of the combinations of the collection dates showed significant differences. Using ANOSIM, we did not observe significant differences in bacterial community composition between 7 March/1 April, 7 March/18 April, and 7 March/4 May, and ANOSIM R values were generally low and overcame 0.25 only for the most distant communities, i.e., 19 February/4 May.

By pairwise comparisons of the relative abundances of taxa across collection dates using the Wilcoxon test, we further analysed the fluctuations of the community of core bacterial taxa (occurring in >50% samples and relative abundance >1%) mature cherry laurel leaves collected at different dates which correspond to the transition period from winter to spring season (Table 2). Individual bacterial taxa showed different patterns of relative abundance fluctuations. The relative abundance of Actinobacteria significantly decreased (*p* = 0.020) in late spring (4 May) as compared with mid-spring collection dates (1 April and 18 April). A decrease in late spring was also observed for the most abundant Corynebacterium sp. ASV 0003 and the family Micrococcaceae, although the fluctuation pattern throughout the sampling period differed. Firmicutes and Proteobacteria showed no difference in the relative abundance between late winter (19 February) and late spring (4 May). There was a transient increase in the relative abundance of Firmicutes in the middle collection dates, while the opposite pattern was observed for Proteobacteria. In Proteobacteria, the relative abundance of Alphaproteobacteria decreased with the onset of spring, while Gammaproteobacteria showed an opposite pattern. In contrast, Betaproteobacteria did not show significant changes in relative abundance throughout the sampling period. At the phylum level, Bacteroidetes did not show significant shifts in relative abundance among the collection dates. Chryseobacterium did show a significant increase between late winter and late spring, while Epilithonimonas only showed a significant change between 18 April and 4 May.

**Figure 6:**
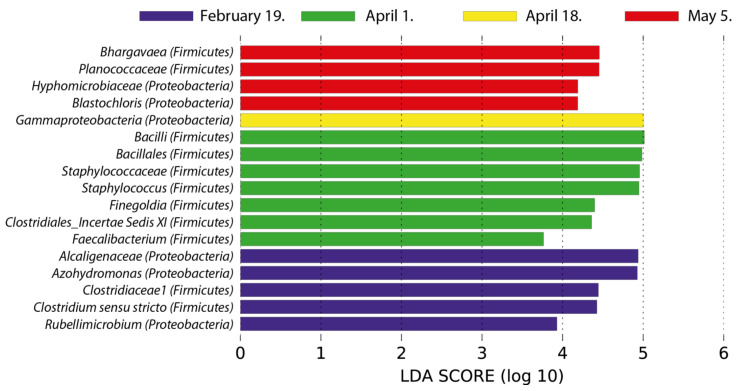
Biomarker taxa analysis of bacterial endophytic community members in mature P. laurocerasus leaves. Histogram shows LDA scores computed for differentially abundant bacterial taxa and identifies which taxa among all those detected as statistically differentially abundant explain the greatest differences between collection dates. No differentially abundant taxa were identified for the early spring (7 March) collection date.

Using biomarker taxa analysis by LEfSe algorithm [40], we identified bacterial taxa, which were differentially abundant in the individual collection dates (Figure 6, Figure S3). Gammaproteobacteria were identified as biomarker taxa in mid-April samples. Some representatives of Firmicutes were identified as biomarker taxa groups in early April samples while minor community members of Firmicutes (Bhargavaea, Planococcaceae) and Alphaproteobacteria (Blastochloris, Hyphomicrobiaceae) were identified as differentially abundant for late spring (4 May). Members of Betaproteobacteria were differentially abundant members of the bacterial community in late winter (19 February).

## 3. Discussion

In this study, we examined the temporal diversity and community composition of core bacteria associated with the leaf endosphere of evergreen cherry laurel shrubs. Sequencing of samples resulted in a lower-than-expected number of high quality sequence reads, which was caused by a relatively high proportion of chimeric and mitochondrial sequences in all but two samples. This certainly lowered the chance to observe rare taxa and might underestimate the bacterial biodiversity to a certain degree. In our case study, we rather focused on the most common community members and how their relative abundance changes during the winter-to-spring transition. Read sampling depths around 1000 sequences are sufficient for providing a picture of the core community composition using DNA metabarcoding in environmental samples, as has been demonstrated in a recent study by Shirazi et al. [41]. Moreover, we obtained saturated rarefaction curves in the vast majority of the leaf samples (Figure S1), which indicates that the impact of the lower sampling depth on bacterial diversity estimates is minimal. Our observation is consistent with common observations of lower numbers of ASVs in surface-sterilized leaves compared to other parts of the plant (e.g., roots) or in the rhizosphere [28].

The four bacterial phyla that dominated the endophytic communities of cherry laurel leaves in this study—*Proteobacteria, Actinobacteria, Firmicutes*, and *Bacteroidete*s—are also typical for the leaf endophytic bacterial communities detected in other temperate woody and herbaceous plant species [21,24,30,31,36,42], suggesting a substantial overlap in the key community members across the host species. In contrast, we did not observe the prominent presence of members of *Acidobacteria* and *Alphaproteobacteria* which comprised the most prevalent bacterial endophytes in the needles of coniferous evergreens *Pinus flexilis* and *Picea engelmannii* growing at high elevation [3,43]. Within the *Proteobacteria, Gammaproteobacteria* were by far the most abundant class in the cherry laurel leaf endophytic community, which is consistent with the observed community composition in the needles of *Pinus radiata* D. Don [44] but not in leaves of evergreen *Magnolia grandiflora*, where *Alphaproteobacteria* dominated the bacterial phyllospheric community. This might be caused by the fact that, in the *Magnolia* study, the phyllospheric bacterial community has been studied, which might differ significantly from the endophytic community [45]. Domination of *Actinobacteria* classes has also been observed in Chinaberry tree tissues (*Melia toosendan*) [46]. Similar to our results, Zhao et al. found that, in Chinaberry tree tissues, *Corynebacteria*-associated taxa were approximately six times more abundant than *Micrococcus*-associated taxa [46]. *Corynebacterium*, *Chryseobacerium*, and *Acinetobacter* were the most abundant genera in our samples. Bacteria from the genus *Corynebacterium* are Gram-positive bacteria, which have been isolated from various habitats including soil, water, animals, and plants [47]. They have been shown to asymptomatically colonize tissues of rice [48], sugar beet [49], citrus plants (*Citrus sinensis* (L.) Osbeck) [50], and rambutan (*Nephelium lappaceum* L.) [51]. *Corynebacterium* representatives have been shown to possess antagonistic activity against several phytopathogens, including *Xanthomonas campestris, Pseudomonas, Helminthosporium, Cercospora, Plasmodiophora brassicae*, and *Ralstonia solanacearum* [51]. A few of the *Corynebacterium* species have also been described as human and animal pathogens [52]. The genus *Chryseobacterium* contains Gram-negative bacteria that have been isolated from water and soil and can be associated with plants [53]. *Chryseobacterium* members were found as endophytic bacteria in corn [54], coffee bean [55], and cucumber (*Cucumis sativus* L.) [56]. *Chryseobacterium* spp. has been shown to produce plant hormone auxin, aminocyclopropane-1-carboxylate deaminase, siderophore, and show antifungal activity [57]. *Acinetobacter* sp. strains are known to utilize diesel fuel and other recalcitrant organics such as carbon and energy sources [58] and were isolated as endophytes from poplar (*Populus trichocarpa* Torr. and A. Gray ex Hook.) [59], willow (*Salix sitchensis* Sanson ex Bong.) [60], or the herb *Commelina communis* L. [61]. *Acinetobacter* spp. have been shown to possess diazotrophic activity, increasing the nitrogen supply to plants, thus enhancing plant growth under nitrogen limitation [60], and representatives of this genus are also known for their potent phyto-remediating activities [62,63]. The abundant presence of these microbes in cherry laurel leaf tissues indicates that this plant might be an interesting novel source of microbes with plant growth-promoting, biocontrol, and/or biosynthetic properties.

The evergreen nature of broadleaf cherry laurel enabled us to study the shifts in the diversity and composition of bacterial endophytic communities in fully developed leaves during the transition period from winter dormancy to vegetative growth. We observed that the relative abundance of the predominant bacterial phyla was highly variable among collection dates, shrubs, and also within individual *P. laurocerasus* shrubs. At the genus level, the relative abundance of dominant bacteria also varied greatly among individual samples (leaves). High variability of endophytic ASVs could possibly be caused by a sporadic and uneven colonization pattern of the aerial plant compartments of cherry laurel by bacterial endophytes, as has been shown previously in poplar trees [30]. This is in contrast with the study performed by Jackson and Denney [36], who found minimal leaf to leaf variation in the phyllospheric bacterial community over time in Southern Magnolia. However, these authors used denaturing gradient gel electrophoresis for the profiling of the phyllospheric bacterial community, which might underestimate the overall diversity [64].

Our data showed variation in the ASV richness, evenness, and diversity estimates of bacterial endophytes in the leaf endosphere during the transition from winter to late spring. The ASV richness and diversity estimates were significantly higher in the samples taken in May than those from other months. The effect of the sampling season on alpha diversity and community composition was also demonstrated in other plant species. The correlation of bacterial diversity with the season has also been observed in grape vine [65,66] as well as elm endosphere [67].

We observed a high variation in the composition of bacterial endophytic communities in leaves between collection dates, as suggested by PERMANOVA, pair-wise comparisons, the NMDS analyses, the relative abundance of bacterial classes, proportions of shared and unique OTUs between sampling months, and the LEfSe analysis. Strong seasonal effects on the endophyte community structures have been also reported in studies on the endophytes of urban trees of *Acer negundo* L., *Ulmus pumila* L., and *Ulmus parvifolia* Jacq. [33], the endophytes of maple tree sap (*Acer saccharum* Marsh.; [34] and buds of Scots pine trees (*Pinus sylvestris* L.) [35]. Surprisingly, however, variation in cherry laurel bacterial endophytic community was reflected only by a few core community members, suggesting that the cherry laurel core endophytic community is relatively stable during the winter/spring seasonal transition and that the differences are mostly associated with the lower abundant community members. Together with the ASV richness, Pielou´s evenness and Shannon´s diversity estimates that these results indicate that, with the start of the vegetation season, there is an increase in the level of new microbial infections of leaves, possibly due to an increased number of opportunistic infections by facultative or passive endophytes. These might originate from different sources such as airborne particles [68], insect vectors [69], or rhizosphere (bacterial endophytes systematically colonizing plant tissues) [30]. Upon the warming and photoperiod lengthening, the remobilization of resources and restoration of growth occurs in evergreen perennials, and are associated with changes in leaf physiology, resource allocation, and chemical composition [70,71,72]. All of these factors could also have an impact on microbial diversity and the community composition in leaves. The relatively stable community composition of core taxa throughout our study period is consistent with other studies on evergreen trees [3,29].

DNA metabarcoding allows for the analysis of microbial communities without cultivation and thus also covers noncultivable bacterial species. However, this approach suffers from common problems, such as chimera formation and sequencing errors, and the outcomes are highly depended on software analysis [73]. Because of this, it is always desired to confirm the presence and diversity of bacterial endophytes through another method such as cultivation analysis or fluorescent in situ hybridization [74]. Further research is therefore needed to draw a complete and accurate picture of the whole endophytic bacterial community in *P. laurocerasus* leaves.

## 4. Materials and Methods

### 4.1. Study Site, Sampling and Processing of Samples

A total of four seed-grown mature shrubs of cherry laurel (*P. laurocerasus* L., intraspecies hybrid of cv. ‘Rotundifolia’), designed as “B”, “D”, “E”, “F” with approximately the same height (2.5–3 m) were marked for sampling in the area of Mlynany Arboretum, Vieska nad Zitavou, Slovakia (48°19’10” N, 18°22’07” E). The distance between individual shrubs (each representing a biological replicate) was at least 50 m. Sampling dates were chosen to cover the period of transition between the winter and spring seasons: late winter (19 February), early spring (7 March), mid spring (1 April, 18 April), and late spring (4 May). Three fully developed leaves with no disease symptoms were collected from the middle part of different one-year-old branches on the same shrub at approximately the same height of 1.5 m. Leaves were placed within sterile sample plastic bags and kept on ice until processing (within four hours of collection). Each collected leaf was processed separately and represented a technical replicate. Meteorological data on temperature and precipitation during the sampling period were recorded by the weather station located in Mlynany Arboretum (Figure S2). Plant surface sterilization procedure was similar to that used by [75]. It included the following steps: first, to wash out the mechanical impurities, leaves were thoroughly washed under tap water, then in sterile distilled water, and were surface sterilized in 2.5% (*v*/*v*) sodium hypochlorite solution for 5 min, followed by a final wash in sterile distilled water for 1 min. The efficacy of the sterilization procedure was validated by plating an aliquot of the rinse water on nutrient agar. Three leaf discs of 14 mm in diameter (about 0.05 g each) were cut off from the middle part of the leaf blade (the middle vein and leaf margins were excluded) using a sterilized corkborer and were transferred to a sterilized 2 mL microcentrifuge tube. Samples were frozen and kept at −80 °C until further processing.

### 4.2. DNA Extraction, PCR Conditions and Library Construction

The deep frozen plant material was homogenized to a fine powder by shaking with yttrium stabilized zirconium oxide beads (5 mm diameter) using TissueLyser II (Qiagen GmbH, Hilden, Germany) at maximum frequency (30 Hz) for 2 min. DNA was extracted using the MO BIO’s Powersoil DNA Isolation Kit (Qiagen GmbH, Hilden, Germany) following the provided protocol. The quality of the isolated DNA was tested on 1% (*w*/*v*) agarose gel before use as a template in PCR. Bacterial chloroplast-excluding primers 515F [76] and 799R, i.e., reversed 799F [77], were used for amplification of the V4 region of the 16S rRNA gene. On 5’ side, primers were enhanced by 6 nt sequence (taq), which allowed for the identification of a sample in the pooled library, followed by 0, 1, or 2 nt for the increasing diversity of reads, and 2 nt spacer to separate original primer sequence [78]. The composition of the 30 µL PCR mixture was as follows: 20 ng of DNA, 0.3 µM of each primer, and 15 µL of a KAPA HiFi HotStart ReadyMix (2X) reaction buffer (Kapa Biosystems, Wilmington, MA, USA). Ultrapure demineralized water (1 µL) was used instead of DNA template for negative control PCR. DNA aliquots were PCR-amplified in a SureCycler 8800 thermal cycler (Agilent technologies, Santa Clara, CA, USA) with 90 s denaturation at 98 °C, 35 cycles of 15 s denaturation at 98 °C, 15 s annealing at 62 °C, and 15 s elongation at 72 °C, after which a final elongation step of 120 s at 72 °C was performed. The PCR products were visualized on agarose gels (2% in TBE buffer) containing ethidium bromide and purified with the PCR Purification Kit (Jena Bioscience). The PCR product concentration was measured on the Qubit 2.0 Fluorometer using the Qubit dsDNA HS Assay Kit (Thermo Fisher Scientific, Waltham, MA, USA). DNA was adjusted to an equal concentration and pooled together. Illumina adapters were attached by the Truseq LT PCR-free kit (Illumina, San Diego, CA, USA) with a modification involving the skipping of the DNA fragmentation and size selection steps. The library was quantified by qPCR using the NebNext Quantification kit (New England Biolabs, Ispwich, MA, USA), diluted to 4 nM concentrations and denatured. The MiSeq Reagent Kit v3 (600-cycle) was used for sequencing. Finally, 600 µL of 20 pM library with 1% PhiX spike was loaded into the cartridge.

### 4.3. Sequence Processing

Acquired sequence data were processed in the SEED2 ver. 2.1 software [79]. Using this software, sequences were joined, assigned to individual samples according tag sequences, and primers were trimmed. Sequences with an overall quality lower than Q30 were removed from further analysis. Moreover, sequences with length below 250 bp and above 350 bp were discarded. QIIME 2 ver. 2020.6 [80] was used for denoising and the generation of amplicon sequence variants (ASVs) with DADA 2 algorithm [81]. ASVs were identified using the Ribosomal Database Project (RDP) Classifier ver. 2.13. [82]. ASVs that were identified as chloroplasts and mitochondria were removed from further analysis. 

### 4.4. Community Structure Composition, Temporal Variation of Individual Community Members, and Analysis of Differential Abundance

Temporal variation of the core taxa at each taxonomic level (phylum to ASV) was expressed as difference in their relative abundances between collection dates. Firstly, relative abundances (RA) of all taxons in each sample were calculated for each taxonomic level (phylum to ASV). For each taxon, pairwise comparisons across all sampling dates were done by unpaired Wilcoxon test in R [83]. Significance was expressed by letter labels assigned according resulting *P*-values using multcompview package [84] in R. The most commonly identified genera were visualized by heatmap generated in Heatmap3 package [85] in R. samples, i.e., shrubs (columns) as well as genera (rows) were clustered by complete-linkage clustering based on Bray–Curtis dissimilarity. To visualize the number of shared and unique ASVs between sampling dates, data for Venn diagrams were calculated using MS Excel and visualized using venn package [86] in R. LEfSe analysis [40]. Galaxy server (https://huttenhower.sph.harvard.edu/galaxy/; accessed on 5. 1. 2022) was used for detection of biomarker taxa for sample collection dates. All the aforementioned analyses were done on a nonrarefied sequence dataset.

### 4.5. Analyses of Diversity

Shannon’s diversity index, Pielou’s corrected evenness, ASV richness, and weighted UniFrac distance matrix [87] were calculated in QIIME 2 after rarefaction to the lowest observed reads per sample value (889 sequences) (Figure S1). Variation in these diversity indices was analysed using ANOVA followed by the Tukey test in R environment. Normality of ANOVA residues and the equivalence of variances was confirmed by Shapiro–Wilk and Levene tests, respectively. UniFrac distance matrix was used for nonmetric multidimensional scaling (NMDS) analysis using Vegan package [88] in the R environment. Factors affecting microbial community were analysed by UniFrac distance-based Permutational Multivariate Analysis of Variance Using Distance Matrices (PERMANOVA) in the Vegan package. Homogeneity of dispersion was analysed using betadisper function in the Vegan package. Permutation test for homogeneity of multivariate dispersions resulted in P-values higher than 0.05 that confirmed the validity of the PERMANOVA result. PERMANOVA pairwise comparisons were made using R package RVAideMemoire [89]. Differences between bacterial community compositions were also evaluated by analysis of similarity (ANOSIM) test.

## 5. Conclusions

Our case study explored the diversity and community structure of bacterial endophytes in *P. laurocerasus* using Illumina amplicon sequencing. We revealed that the cherry laurel leaf endosphere was predominantly inhabited with members of *Corynebacterium*, *Chryseobacerium*, and *Acinetobacter.* The composition of the core community of bacteria and archaea in cherry laurel leaf endosphere only partially changed during the transition from winter dormancy to vegetative growth. In the late spring, the diversity indices significantly increased in comparison to winter/early spring months, possibly due to an increased number of opportunistic infections by facultative or passive endophytes. The study of microbial community dynamics during the whole lifespan of *P. laurocerasus* leaves is desirable to obtain a more detailed picture of the environmental factors affecting its associated microbial community.

## Figures and Tables

**Table 1:** Sampling date effect on bacterial community structures. For calculation, a pairwise comparison using PERMANOVA on a weighted UniFrac distance matrix was used.

Collection date	19 February	7 March	1 April	18 April
19 February				
7 March	0.002			
1 April	0.001	0.162		
18 April	0.002	0.018	0.002	
4 May	0.001	0.069	0.004	0.014

**Table 2:** Fluctuations of the *Prunus laurocerasus* L. endophytic bacterial microbiome during the seasonal transition from winter dormancy to vegetative growth in fully developed leaves. Only core taxa, i.e., taxa occurring in >50% samples and with relative sequence abundance >1% are listed. The relative abundance (RA) of a given taxon is the number of sequences associated with that taxon over the total number of sequences in the dataset. Significant differences among collection dates according to Wilcoxon test (*p* < 0.05) are indicated in lower-cased letters.

Phylum						Average RA percent (number of positive samples)p-value					Mean RA of all samples (%)
	Class					19.2.	7.3.	1.4.	18.4.	4.5.	
		Order									
			Family								
				Genus							
					ASV						
**Actinobacteria**						**19.2 (12)ab**	**24.0 (12)ab**	**27.7 (12)a**	**26.2 (12)a**	**15.3 (12)b**	**22.5**
	*Actinobacteria*					**19.2 (12)ab**	**24.0 (12)ab**	**27.7 (12)a**	**26.2 (12)a**	**15.3 (12)b**	**22.5**
		*Actinomycetales*				18.9 (12)a	23.8 (12)a	27.0 (12)a	22.5 (12)a	14.4 (12)a	21.3
			Corynebacteriaceae			13.2 (10)a	16.2 (11)a	16.0 (12)a	12.7 (12)a	8.5 (8)a	13.3
				*Corynebacterium*		13.2 (10)a	16.2 (11)a	16.0 (12)a	12.7 (12)a	8.5 (8)a	13.3
					0003	4.5 (7)ab	6.5 (11)a	10.0 (11)a	6.4 (10)ab	2.9 (6)b	6.0
			Micrococcaceae			2.4 (7)ab	4.8 (10)a	1.7 (7)ab	2.2 (11)a	1.1 (6)b	2.4
**Bacteroidetes**						**11.0 (12)a**	**14.6 (12)a**	**11.3 (12)a**	**14.7 (12)a**	**15.6 (12)a**	**13.4**
	*Flavobacteriia*					**8.8 (11)ab**	**10.7 (12)ab**	**8.6 (11)a**	**10.5(12)ab**	**13.9 (12)b**	**10.5**
		*Flavobacteriales*				8.8 (11)ab	10.7(12) ab	8.6 (11)a	10.5(12)ab	13.9 (12)b	10.5
			Flavobacteriaceae			8.8 (11)ab	10.7 (12)ab	8.6 (11)a	10.5(12)ab	13.9 (12)b	10.5
				*Epilithonimonas*		3.2 (9)ab	2.6 (9)ab	2.8 (9)ab	1.1 (8)a	4.4 (12)b	2.8
					0008	3.2 (9)ab	2.6 (9)ab	2.8 (9)ab	1.1 (8)a	4.4 (12)b	2.8
				*Chryseobacterium*		5.0 (10)a	6.2 (12)ab	5.8 (10)ab	7.7 (12)ab	9.4 (12)b	6.8
					0002	3.9 (10)a	5.4 (12)ab	5.4 (10)ab	6.9 (11)ab	8.6 (12)b	6.0
**Firmicutes**						**17.2 (12)ab**	**30.6 (12)ac**	**27.7 (12)c**	**16 (12)b**	**17.6 (12)ab**	**21.8**
	*Bacilli*					**14.7 (12)ab**	**22.1 (12)ab**	**20.6 (12)a**	**13.2 (12)b**	**13.5 (12)b**	**16.8**
		*Bacillales*				4.8 (9)a	5.5 (12)ab	8.2 (12)b	7.1 (12)ab	4.6 (12)ab	6.1
			Staphylococcaceae			4.2 (8)ab	4.2 (12)ab	7.0 (12)a	4.8 (12)ab	3.1 (11)b	4.7
				*Staphylococcus*		4.1 (8)ab	4.1 (12)ab	6.9 (12)a	4.8 (12)ab	3.1 (11)b	4.6
					0006	4.1 (8)ab	4.1 (12)ab	6.9 (12)a	4.8 (12)ab	3.1 (11)b	4.6
		*Lactobacillales*				9.9 (12)ab	16.6 (12)a	12.4 (12)a	6.0 (12)b	8.9 (12)ab	10.8
			Streptococcaceae			7.3 (11)a	15.0 (12)a	11.3 (12)a	3.7 (12)b	8.1 (12)a	9.1
				*Lactococcus*		4.3 (11)ab	7.8 (12)ab	7.0 (12)a	2.5 (12)b	6.2 (12)a	5.5
					0004	4.2 (11)ab	7.6 (12)a	6.7 (12)a	2.3 (12)b	6.1 (12)a	5.4
				*Streptococcus*		3.0 (11)ab	7.2 (12)a	4.3 (12)a	1.3 (9)b	2.0 (12)b	3.6
					0014	0.7 (7)ab	4.7 (12)a	1.7 (9)ab	0.8 (8)b	0.5 (7)b	1.7
	*Clostridia*					**2.5 (9)a**	**8.5 (12)ab**	**7.0 (12)b**	**2.6 (12)a**	**3.9 (11)a**	**4.9**
		*Clostridiales*				2.5 (9)a	8.5 (12)ab	7.0 (12)b	2.6 (12)a	3.9 (11)a	4.9
		Clostridiales_Incertae Sedis XI				1.0 (7)a	2.9 (11)ab	3.3 (12)b	2.1 (10)ab	0.9 (11)a	2.0
**Proteobacteria**						**49.8 (12)ab**	**28.6 (12)ac**	**22.6 (12)c**	**38.4 (12)a**	**47.7 (12)b**	**37.4**
	*Alphaproteobacteria*					**13.1 (12)ab**	**2.9 (10)ac**	**2.5 (11)c**	**8 (12)b**	**3.9 (12)c**	**6.1**
		*Rhizobiales*				1.1 (9)a	0.9 (9)a	1.1 (9)ab	4.6 (11)c	2.2 (12)bc	2.0
		*Sphingomonadales*				2.7 (10)a	0.8 (9)ab	0.9 (8)b	2.3 (9)ab	0.9 (8)b	1.5
			Sphingomonadaceae			2.6 (10)a	0.8 (9)ab	0.9 (7)b	1.9 (9)ab	0.9 (8)b	1.4
		*Sphingomonas*				1.8 (9)a	0.6 (9)a	0.9 (7)a	1.3 (7)a	0.8 (8)a	1.1
	*Betaproteobacteria*					**12.8 (12)a**	**5.7 (12)a**	**5.5 (12)a**	**7.3 (12)a**	**9.8 (12)a**	**8.2**
		*Burkholderiales*				11.9 (12)a	4.7 (12)ab	3.9 (12)b	4.4 (12)ab	5.7 (12)ab	6.1
			Comamonadaceae			2.6 (11)ab	3.5 (12)a	2.9 (12)ab	3.5 (12)ab	1.7 (12)b	2.9
				*Delftia*		1.5 (9)ab	2.6 (12)a	1.7 (12)ab	2.8 (10)ab	1.4 (11)b	2.0
					0016	1.5 (9)ab	2.6 (12)a	1.7 (12)ab	2.8 (10)ab	1.4 (11)b	2.0
	*Gammaproteobacteria*					**23.9 (12)ab**	**19.9 (12)ab**	**14.5 (12)a**	**23.0 (12)b**	**33.9 (12)c**	**23.0**
		*Enterobacteriales*				3.1 (12)ab	3.6 (11)ab	2.4 (10)a	3.9 (12)ab	4.8 (12)b	3.6
			Enterobacteriaceae			3.1 (12)ab	3.6 (11)ab	2.4 (10)a	3.9 (12)ab	4.8 (12)b	3.6
				*Enterobacter*		2.4 (12)ab	2.2 (9)ab	1.6 (10)a	3.2 (12)ab	4.0 (12)b	2.7
					0010	2.4 (12)ab	2.2 (9)ab	1.6 (10)a	3.2 (12)ab	4.0 (12)b	2.7
		*Pseudomonadales*				16.6 (12)ab	13.4 (12)ab	10.1 (12)a	11.6 (12)a	19.2 (12)b	14.2
			Moraxellaceae			11.6 (12)ab	8.5 (12)ab	6.6 (12)a	6.8 (12)a	14.1 (12)b	9.5
				*Acinetobacter*		8.7 (12)ab	7.2 (12)a	3.9 (11)b	6.3 (12)ab	8.5 (12)a	6.9
					0007	6.0 (10)ab	3.8 (12)a	1.3 (9)b	3.7 (11)ab	4.1 (12)a	3.8
					0015	1.8 (12)a	2.2 (10)a	1.9 (9)a	1.7 (7)a	1.4 (8)a	1.8
			Pseudomonadaceae			5.0 (12)a	4.9 (12)a	3.3 (12)a	4.8 (12)a	5.1 (12)a	4.6
				*Pseudomonas*		5.0 (12)a	4.9 (12)a	3.3 (12)a	4.8 (12)a	5.1 (12)a	4.6
					0017	2.9 (10)ab	2.1 (12)a	1.6 (10)ab	1.1 (9)b	2.8 (10)ab	2.1
					0021	1.4 (8)a	1.3 (11)a	0.8 (8)a	1.4 (11)a	1.3 (12)a	1.2
		*Xanthomonadales*				3.6 (10)abc	2.1 (10)ab	1.7 (11)a	5.7 (11)bc	6.3 (12)c	3.9
			Xanthomonadaceae			3.6 (10)ab	2.1 (10)a	1.7 (11)a	5.5 (11)ab	5.9 (12)b	3.8
				*Stenotrophomonas*		3.1 (8)ab	1.9 (10)a	1.4 (10)a	4.5 (9)ab	4.9 (12)b	3.2
					0011	3.1 (8)ab	1.8 (9)a	1.4 (10)a	2.8 (9)ab	4.8 (12)b	2.8

## Data Availability

The nucleotide sequence data reported are available in the GenBank databases under the BioProject ID: PRJNA609065.

## Supplementary Materials

The following are available online at https://www.mdpi.com/article/10.3390/plants11030417/s1, Figure S1: Rarefaction curves for all samples. Figure S2: Graph of temperature and precipitation in the sampling period. Figure S3: Biomarker taxa analysis by LEfSe algorithm. Table S1. Sampling date effect on bacterial community structures calculated using ANOSIM.
