# Supplementary material for: Changes of Endophytic Bacterial Community in Mature Leaves of Prunus laurocerasus L. during the Seasonal Transition from Winter Dormancy to Vegetative Growth
Source: Plants (Basel). 2022 Feb 3;11(3):417. doi: 10.3390/plants11030417 (PMC8839770; doi:10.3390/plants11030417)
Supplement: Supplementary file 1 [file plants-11-00417-s001.zip › Table S1.pdf]

Table S1. Sampling date effect on bacterial community structures calculated using ANOSIM. *p*-values are given in the upper half of the table; ANOSIM R values are given in the lower half of the table; Significant comparisons are shown in bold. Significance levels: * $p \leq 0.05$; ** $p \leq 0.01$.

Collection Date	19 February	7 March	1 April	18 April	4 May
19 February	NA	0.005 **	0.002 **	0.009 **	0.001 **
7 March	0.166561	NA	0.178	0.056	0.327
1 April	0.196444	0.028725	NA	0.004 **	0.044 *
18 April	0.154882	0.07218	0.135732	NA	0.021 *
4 May	0.291298	0.015993	0.085122	0.118056	NA
