# Supplementary figures and images for: Changes of Endophytic Bacterial Community in Mature Leaves of Prunus laurocerasus L. during the Seasonal Transition from Winter Dormancy to Vegetative Growth
Source: Plants (Basel). 2022 Feb 3;11(3):417. doi: 10.3390/plants11030417 (PMC8839770; doi:10.3390/plants11030417)

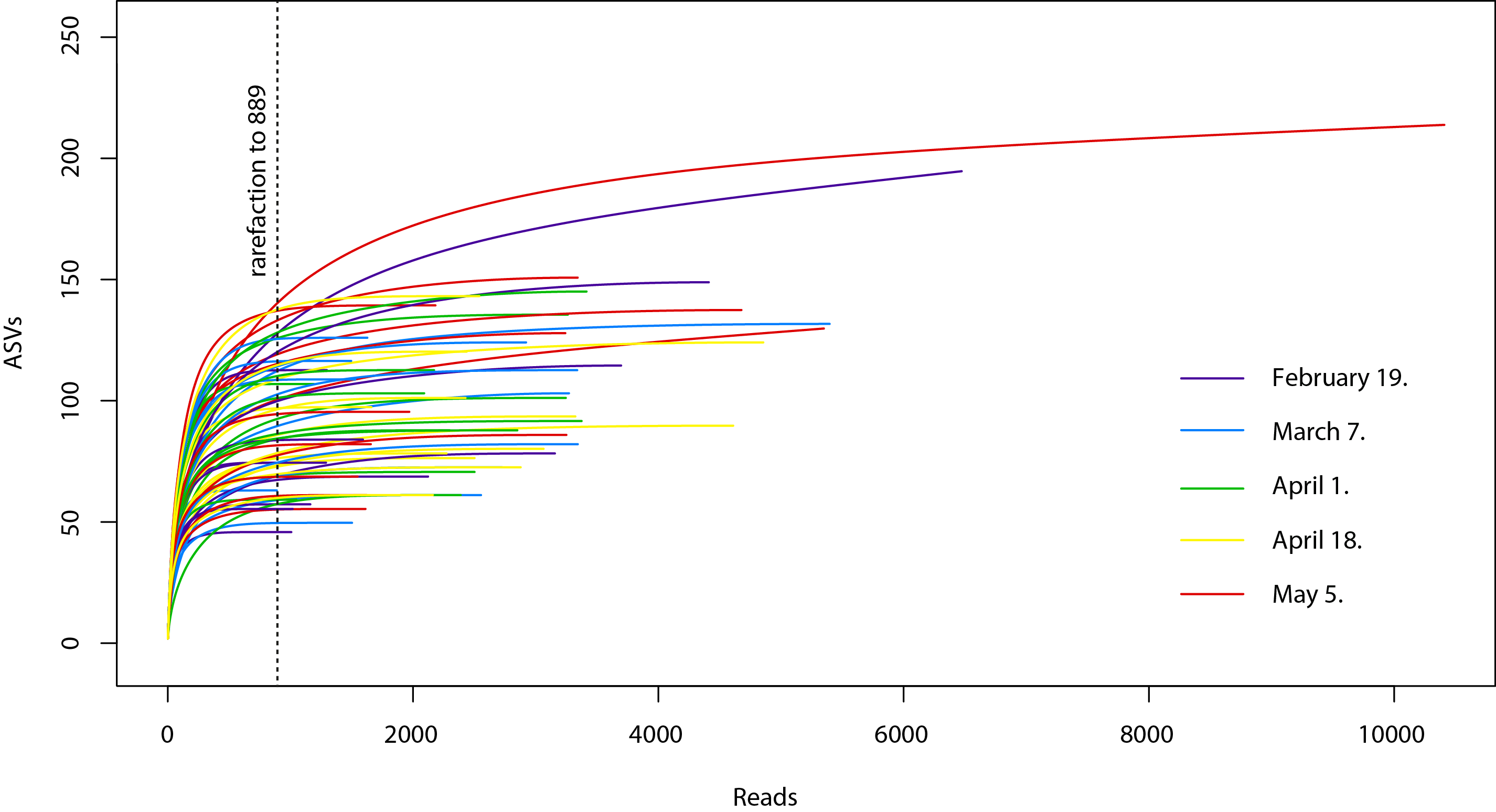

Supplement: Supplementary file 1 [file plants-11-00417-s001.zip › FigureS1.png]

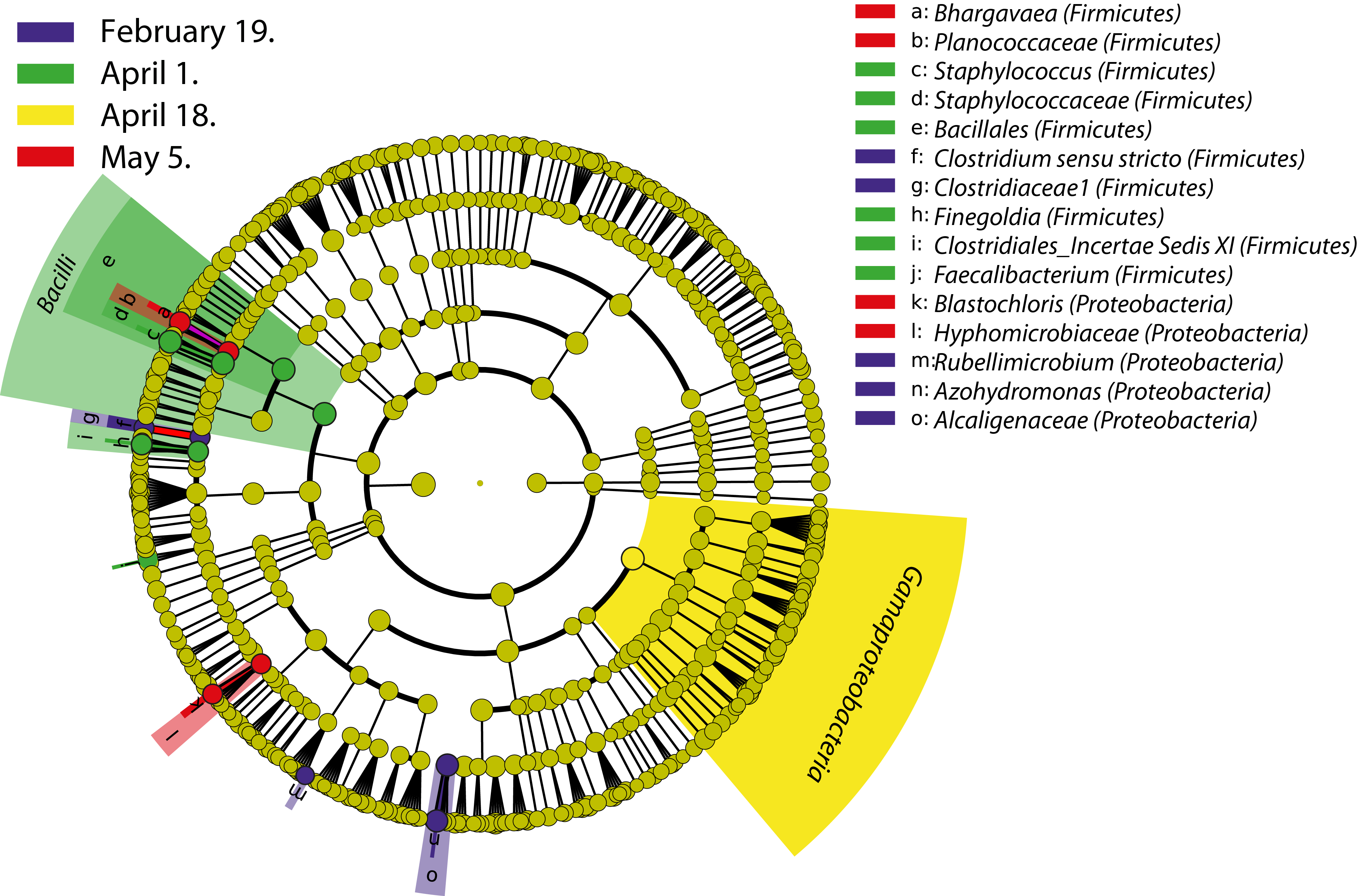

Supplement: Supplementary file 1 [file plants-11-00417-s001.zip › FigureS3.png]

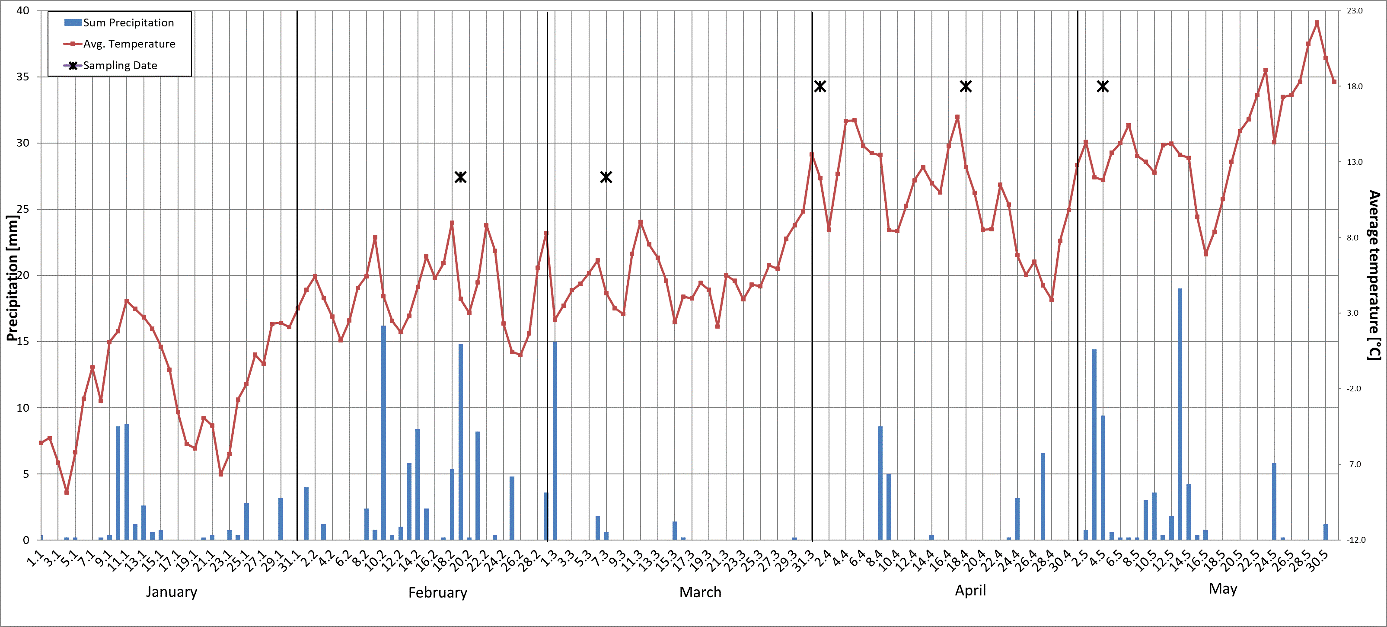

Supplement: Supplementary file 1 [file plants-11-00417-s001.zip › Figure_S2.png]
